# From pelvic geometry to modal compliance: sex-specific deformation pathways in the human pelvic ring

**DOI:** 10.1098/rsif.2026.0555

**Published:** 2026-10-07

**Authors:** Michal Kuchař, Alexander Morávek, Niels Hammer, Petr Henyš

**Affiliations:** ^1^ Department of Anatomy, Faculty of Medicine in Hradec Králové, Charles University, Hradec Králové, Czech Republic; ^2^ Division of Macroscopic and Clinical Anatomy, Gottfried Schatz Research Center for Cell Signaling, Metabolism and Aging, Medical University of Graz, Graz, Austria; ^3^ Department of Orthopedic and Trauma Surgery, University of Leipzig, Leipzig, Germany; ^4^ Institute of New Technologies and Applied Informatics, Faculty of Mechatronics, Informatics and Interdisciplinary Studies, Technical University of Liberec, Liberec, Czech Republic

**Keywords:** biomechanics, human pelvis, modal analysis, finite-element modelling, childbirth, allometry, sacroiliac joint

## Abstract

Walking, posture and childbirth all depend on the pelvic ring, yet human pelvic dimorphism is usually interpreted from static birth-canal dimensions. We asked whether sex differences in pelvic function are explained by overall size and static pelvimetry or by available mechanical deformation pathways. We analysed 278 anatomically standardized lumbopelvic finite-element models built from clinical computed tomography scans. In each model, modal decomposition separated pelvic deformation into a shared, ranked set of pathways, allowing us to compare pathway stiffness, load recruitment and sensitivity to ligamentous and cartilaginous constraints. Overall pelvic size accounted for much of the baseline stiffness spectrum, confirming a strong size-dependent component. However, size- and age-adjusted sex differences remained: male pelves showed approximately 20% higher eigenvalues than female pelves in modes 2 and 9. Loading probes showed a common backbone in both sexes during habitual support, whereas inlet opening revealed a different response organization, with female pelves distributing deformation across a broader, coordinated set and a higher effective number of participating modes. The principal inlet-opening pathway was most sensitive to the pubic symphysis and anterior sacroiliac ligamentous constraints. Pelvic dimorphism, therefore, lies not only in canal shape, but in the organization of mechanical pathways that balance support with deformation.

## 1. Introduction

The human pelvis sits at the intersection of biomechanics, evolutionary anthropology and obstetrics. It must transmit loads between the trunk and lower limbs while also forming the bony boundary of the birth canal. This dual role has often been framed geometrically: selection for efficient bipedalism is assumed to constrain pelvic form, whereas selection for parturition favours canal enlargement [1–3]. Linear dimensions and canal shape are therefore central to most discussions of pelvic function.

A purely geometric account is incomplete, however, because the pelvis does not behave as a rigid frame. It is a constrained, deformable ring whose response depends on available deformation pathways, the stiffness cost of accessing those pathways and the constraints imposed by the sacroiliac joints (SIJs), pubic symphysis and ligamentous architecture. This distinction matters for both locomotor and obstetric interpretation. Moderately wider hips do not appear to impose a simple locomotor cost [4], while recent syntheses of childbirth evolution emphasize the pelvic floor, connective tissues and broader parturition mechanics rather than bony canal dimensions alone [3,5,6]. Static pelvimetry remains informative, but it is unlikely to capture the full functional phenotype.

Computational modelling provides a route from anatomy to mechanics, especially when heterogeneous bone material, cartilage and ligaments are represented [7–13]. Dynamic birth simulations and pelvic finite-element studies have shown that mechanical interpretation can extend beyond static canal shape [14]. Most existing studies, however, are subject-specific or based on small specimen cohorts and therefore do not directly show which mechanical features are shared across individuals and which are primarily allometric [9–11,15].

Here, we address this gap using 278 standardized human pelves in anatomical correspondence. Rather than reducing each pelvis to a set of obstetric diameters, we describe the pelvic ring as a repertoire of elastic deformation pathways. This separates two questions that are usually conflated: which pathways are mechanically accessible, and how strongly a given load recruits them.

Our working hypothesis was that sex differences in pelvic mechanics are not reducible to overall size or to any single canal dimension. Instead, scale should set some of the baseline stiffness spectrum, whereas sex should be expressed in selected pathways and in the way load cases distribute response energy across them. We therefore tested three linked expectations: allometry explains a share of the variation in modal stiffness; selected sex differences persist after adjustment for size and age; habitual support loads and birth-canal-relevant opening probes recruit different parts of the modal repertoire. Support for these expectations would shift the functional interpretation of pelvic dimorphism from static geometry towards mode-specific compliance within a constrained mechanical ring.

## 2. Material and methods

### 2.1. CT-derived cohort in anatomical correspondence

The analysis used BoneDat, a standardized database of 278 clinical lumbopelvic computed tomography (CT) scans from a Central European cohort spanning adolescence to old age [16]. The present dataset comprised 150 female and 128 male individuals aged 16–91 years. Subject selection, segmentation, shape normalization, template construction, mesh generation and technical validation are reported in the source data descriptor [16]; here, we summarize only the elements needed for the mechanical analysis. Access to and further use of the database were approved by the Ethics Committee of University Hospital Hradec Králové (Ref: 202411IO3P), as reported in the source data descriptor [16].

The resource provides curated segmentations, diffeomorphic deformation fields, a common reference surface, tetrahedral volumetric meshes and a template model. This allowed every pelvis to be analysed on a shared anatomical domain while preserving individual morphology through subject-specific deformation fields. Because the original scans were acquired without a calibration phantom, mapped material properties are interpreted as cohort-wise relative descriptors rather than direct clinical densitometry. Phantomless internal calibration was used to convert the released Hounsfield-unit values to hydroxyapatite-equivalent density before elastic property assignment.

### 2.2. Allometric scale and conventional pelvic dimensions

The principal size variable was the cube root of total pelvic volume relative to the template reference volume, $S=(V/V_{\mathrm{template}}{)}^{1/3}$. This gives a whole-pelvis linear scale for a three-dimensional structure and avoids using a single canal diameter as a proxy for body size. We also recorded total surface area A, total mass m, effective thickness $t_{\mathrm{eff}}=V/A$ and effective density $\rho_{\mathrm{eff}}=m/V$, where V denotes total pelvic volume. These effective quantities are bulk descriptors used for cohort-level comparison rather than local measurements of cortical thickness or calibrated tissue density.

Conventional pelvimetric variables were extracted from the registered cohort for comparison with the mechanical measures and to test whether these secondary pelvimetric measures explained modal stiffness. These were anteroposterior inlet diameter (AP), pelvic inlet transverse diameter (PIT), subpubic angle, biacetabular width, biischiadic width, bituberous width, sacral width and iliopectineal eminence width. They were analysed as continuous anatomical comparators, not as categorical pelvic types or as replacements for the modal mechanical descriptors.

### 2.3. Finite-element spectrum as accessible deformation pathways

Each finite-element model started from the same tetrahedral template mesh. The mesh was warped into subject space with the subject-specific BoneDat deformation field, so that corresponding nodes and regions referred to the same anatomy across the cohort while retaining individual pelvic shape. Mechanics were assembled on the mapped reference domain. Bone properties were assigned cell-wise from mapped hydroxyapatite-equivalent density. The sacroiliac cartilage and pubic symphysis were represented as finite-thickness volumetric solid regions, continuously connected to the adjacent bones and assigned region-wise baseline material properties. Here, an interface region denotes an anatomical load-transfer subdomain, not a zero-thickness contact surface or contact algorithm.

The mechanical model was comparative by design. It used small-deformation, linear isotropic elasticity. Rigid support was imposed by penalty constraints, and the major pelvic ligament groups were represented as anatomically oriented linear axial spring bundles attached to corresponding regions. The spring assembly included a prescribed pretension for each ligament bundle through a geometric-stiffness contribution, but remained a reduced linearized representation of ligament behaviour. These choices allowed relative elastic pathway accessibility to be compared across individuals within a common modelling framework. All finite-element matrices and probe responses were assembled and solved using a custom finite-element implementation in DOLFINx 0.10 [17]. FEBio/BoneDat model sources were used to define the tetrahedral mesh and ligament attachment regions, but FEBio was not used as the solver.

For each subject, we asked which deformation patterns were easiest to access within this elastic model. This was done by solving the generalized eigenproblem

(2.1) $$K\phi_{k}=\lambda_{k}M\phi_{k},\;k=1,2,\ldots ,10,$$

where K is the subject-specific stiffness matrix and M is the numerical inner-product matrix used to normalize and compare displacement fields. The modes are quasi-static deformation patterns, not inertial vibration modes. The eigenvalue $\lambda_{k}$ is the elastic cost of the corresponding M-normalized pattern; lower eigenvalues indicate pathways that are more accessible under the adopted metric.

The analyses use modes 1–10. Mode labels were defined after applying the recorded per-subject reordering vectors from the processing pipeline. This step was needed because nearby low-order eigenpairs can exchange numerical order between subjects; after reordering, a label such as mode 9 denotes the aligned deformation family used in the cohort analysis, not merely the ninth raw solver output. The reordering vectors served only for mode correspondence. Because eigenvectors are sign-indeterminate, arbitrary sign changes do not affect the scalar quantities reported here: eigenvalues, modal energy fractions or sensitivity measures.

Two terms are used throughout the manuscript. Pathway accessibility denotes the position of a mode in the stiffness spectrum. Pathway dominance denotes how much of a particular load response is carried by that mode after loading is applied.

### 2.4. Mechanical probes and modal participation

Four static loading scenarios were analysed. Loads were prescribed as vector tractions representing specified total forces and were distributed uniformly over the mapped physical area of each selected surface (figure 1). In every scenario, the superior S1 surface was restrained in all three displacement components by the same penalty formulation, and no symmetry constraint was imposed. Bilateral stance applied 400 N cranially to the left and right acetabular surfaces, whereas unilateral stance applied 800 N cranially to the right acetabular surface. Inlet opening applied equal and opposite 400 N mediolateral forces to the left and right pelvic brim regions (the most lateral portions of the arcuate lines), whereas outlet opening applied equal and opposite 400 N mediolateral forces to the left and right ischial spine surfaces. The stance scenarios represented habitual support mechanics; the opening scenarios were standardized probes with obstetric relevance, not literal simulations of childbirth. The same template-defined regions and loading logic were transferred to each subject-specific model in the custom DOLFINx workflow.

**Figure 1.**
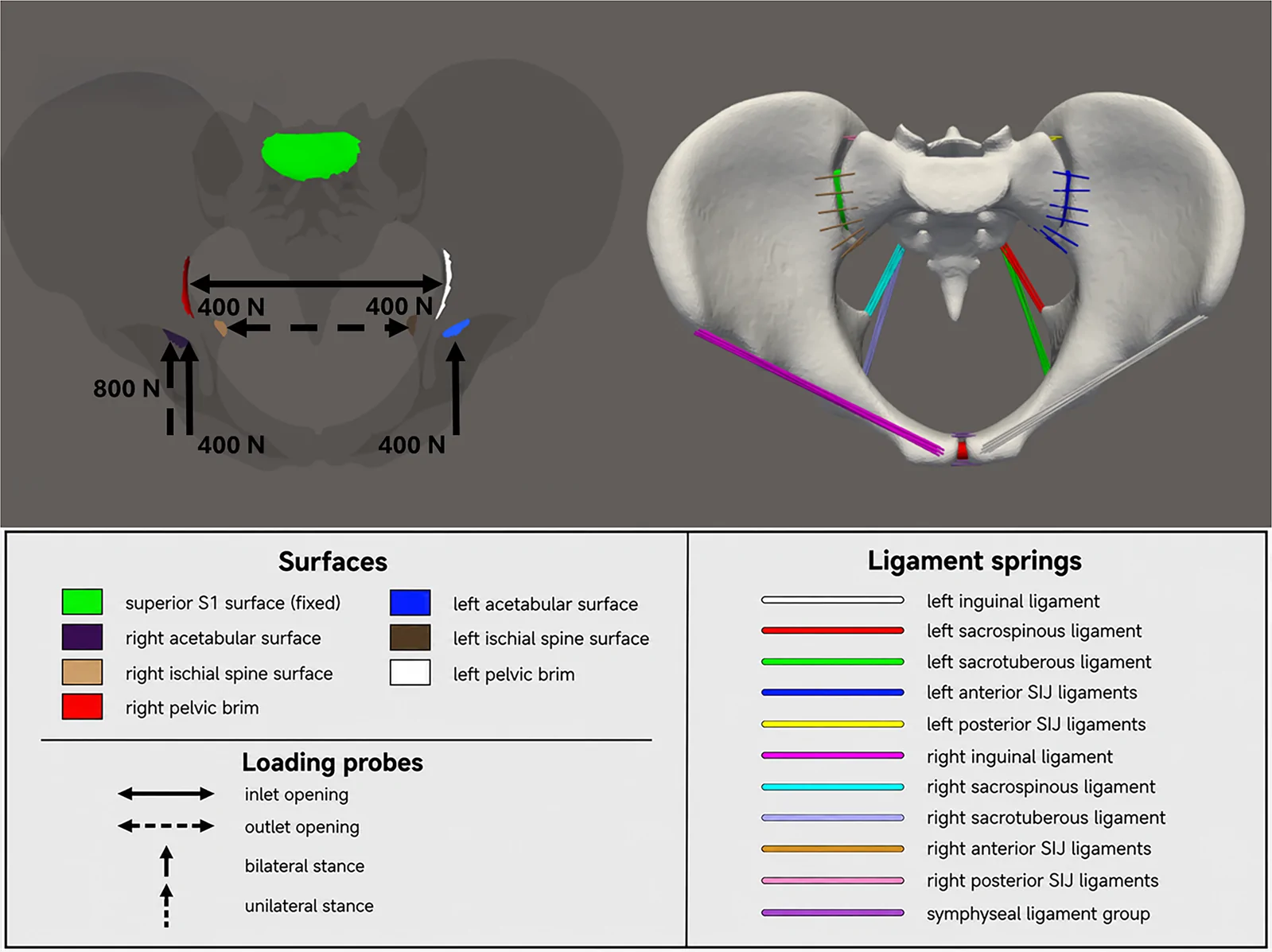
Standardized boundary conditions, loading probes and ligament-spring definitions used in the pelvic finite-element models. The left schematic shows the load-application surfaces and the restrained surface. Both inlet and outlet openings are represented by equal and opposite 400 N mediolateral forces applied to the corresponding left and right loading surfaces. Bilateral stance consists of cranially directed forces of 400 N applied to both acetabular loading surfaces, while unilateral stance uses an 800 N cranially directed force applied to the corresponding right acetabular loading surface. The right schematic shows the linear spring elements representing the pelvic ligaments. Surface and ligament-spring colours correspond to the keys below.

For each subject, the resulting displacement field was represented on the same correspondence-preserving domain as the subject-specific modes and was then projected onto that subject's modal basis. Thus, variation in modal participation reflects both the subject-specific probe response and the subject-specific spectrum, while the anatomical definition of each probe remains standardized. With $\Phi =[\phi_{1},\ldots ,\phi_{10}]$, the participation vector q was obtained from

(2.2) $$(\Phi^{T}M\Phi )q=\Phi^{T}Mu.$$

The modal strain energy associated with each coordinate was then calculated as

(2.3) $$U_{i}=\frac{1}{2}\lambda_{i}q_{i}^{2}\|\phi_{i}{\|}_{M}^{2}\;\text{and}\;\eta_{i}=\frac{U_{i}}{\sum_{j}U_{j}}.$$

The fraction $\eta_{i}$ is the share of the response energy carried by mode i after normalization over modes 1–10. We also tracked the total response energy retained in these 10 modes to check that truncation did not determine the reported patterns.

### 2.5. Local sensitivity of ligament and region constraints

We next asked which soft-tissue constraints most affected the modal spectrum. This was the template-level component of the workflow. For computational tractability, local perturbation operators for ligament stiffness and for the elastic moduli of the sacroiliac cartilage and pubic symphysis regions were prepared once on the population template and evaluated in the common template-referenced coordinate system. For each selected mode, we calculated first-order sensitivities of the eigenvalue to small local changes in ligament stiffness or elastic modulus. These local, linearized measures rank how strongly a local stiffening would change the elastic cost of an M-normalized deformation pathway.

For a ligament bundle with axial stiffness $EA_{\ell }$, the modal sensitivity was

$$\frac{\partial \lambda_{i}}{\partial EA_{\ell }}=\frac{\phi_{i}^{T}(\partial K/\partial EA_{\ell })\phi_{i}}{\phi_{i}^{T}M\phi_{i}}.$$

The reported value therefore reflects the contribution of axial spring stiffness in that ligament bundle to the eigenvalue of mode i.

For the sacroiliac cartilage and pubic symphysis solid regions, the code reported a normalized energy-based sensitivity proxy,

$$S_{i,R}=\frac{E_{i}(R)}{E_{R}\phi_{i}^{T}M\phi_{i}},$$

which is proportional under linearization to $\partial \lambda_{i}/\partial E_{R}$. Ligament and solid-region sensitivities were not compared by raw magnitude because they have different units and arise from different perturbation definitions. Percent contributions were therefore calculated separately within the ligament class and within the region class.

The sensitivity analysis covered the symphyseal ligament group, anterior SIJ ligaments, posterior SIJ ligaments, interosseous SIJ ligaments, sacrospinous ligaments and sacrotuberous ligaments, together with the sacroiliac cartilage and pubic symphysis regions. These template-defined sensitivities were used to rank the anatomical constraints associated with selected modes, rather than to model subject-specific tissue perturbations. The primary focus was on modes showing the clearest adjusted sex effects.

### 2.6. Statistical analysis

Sex differences in cohort descriptors were assessed with Welch two-sample t-tests. Empirical allometry of surface area, mass, effective thickness and effective density was evaluated with log–log regressions against pelvic scale.

Eigenvalues were analysed on the raw scale whenever formal inference was required. Size-normalized eigenvalues were used only for descriptive summaries in table 1 and figure 2. For these summaries, each mode was normalized to the cohort geometric-mean pelvic scale, $S_{0}=\exp(\overset{―}{\log S})=0.9568452943$. The mode-specific scaling exponent was estimated from

**Figure 2.**
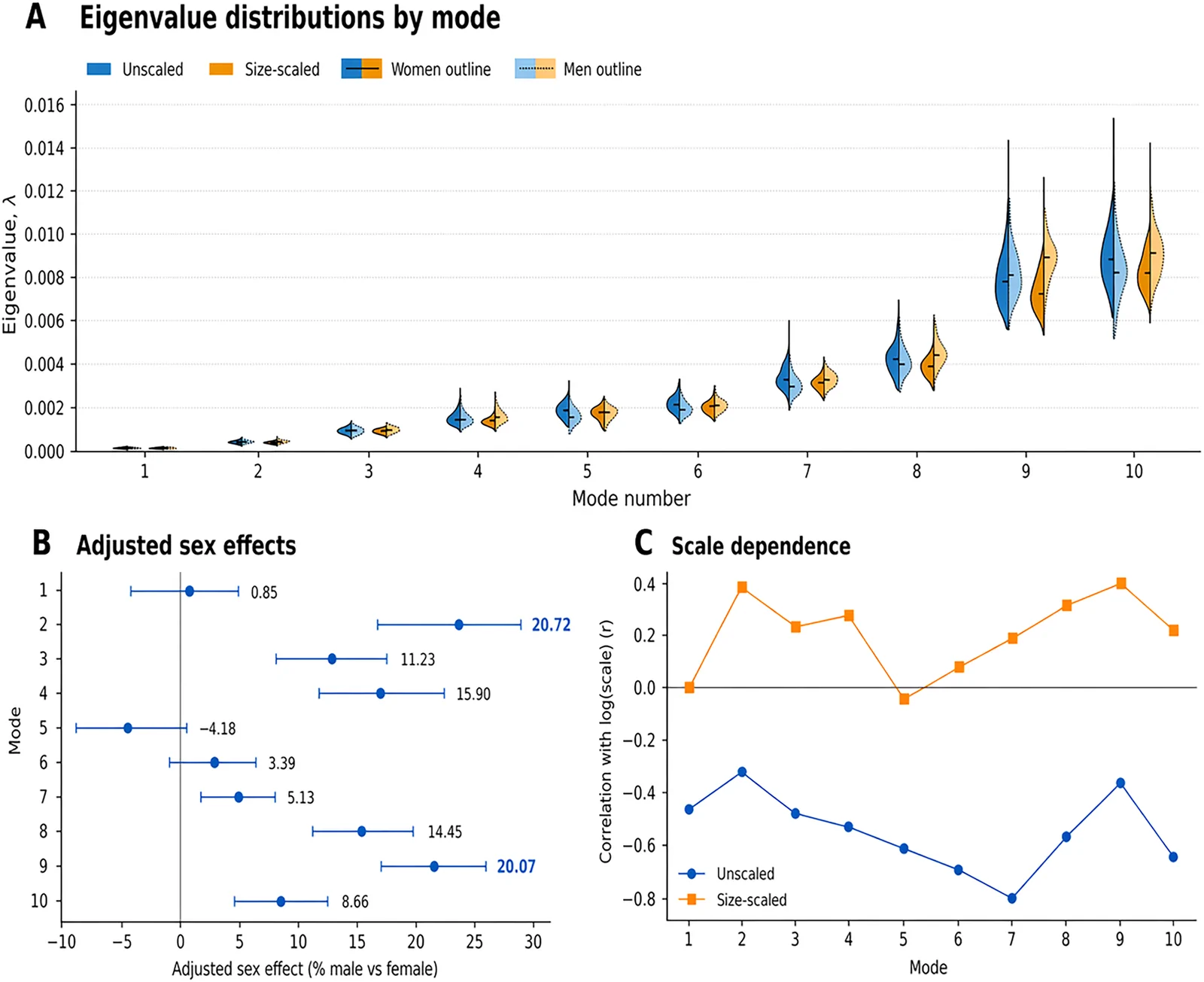
Mode-wise eigenvalue distributions, adjusted sex effects and scale dependence. (A) Distributions of eigenvalues λ for modes 1–10 before and after descriptive allometric size normalization to the cohort geometric-mean scale ($\lambda_{\mathrm{scaled}}=\lambda_{\mathrm{raw}}(S/S_{0}{)}^{\gamma_{k}}$). (B) Adjusted sex effects for each mode, expressed as the percentage difference in eigenvalue in males relative to females after adjustment for pelvic scale and age; points denote estimates and horizontal bars denote the corresponding confidence intervals. (C) Mode-wise Pearson correlations between log⁡(scale) and log⁡(λ) before and after size normalisation.

**Table 1.** Mode-wise scale dependence and adjusted sex effects. Correlations are Pearson's r between log⁡(S) and log⁡(λ) before and after size normalization, $\lambda_{\mathrm{scaled}}=\lambda_{\mathrm{raw}}(S/S_{0}{)}^{\gamma_{k}}$. Sex effects are male-versus-female percentage differences from $\log(\lambda_{\mathrm{raw}})∼\log(S)+\mathrm{sex}+\mathrm{age}$; $q_{\mathrm{sex}}$ values are Benjamini–Hochberg adjusted across modes.

mode	r (unscaled)	p	r (scaled)	p	sex effect	$q_{\mathrm{sex}}$
1	−0.461	$4.96\times 10^{-16}$	0.009	0.876	+0.85% (p=0.734)	0.734
2	−0.331	$1.52\times 10^{-8}$	0.374	$1.20\times 10^{-10}$	+20.72% ($p=2.62\times 10^{-18}$)	$1.31\times 10^{-17}$
3	−0.467	$1.82\times 10^{-16}$	0.233	$8.84\times 10^{-5}$	+11.23% ($p=1.80\times 10^{-7}$)	$3.60\times 10^{-7}$
4	−0.509	$1.04\times 10^{-19}$	0.273	$3.70\times 10^{-6}$	+15.90% ($p=4.81\times 10^{-9}$)	$1.20\times 10^{-8}$
5	−0.607	$2.16\times 10^{-29}$	−0.043	0.480	−4.18% (p=0.106)	0.118
6	−0.684	$9.25\times 10^{-40}$	0.079	0.188	+3.39% (p=0.097)	0.118
7	−0.778	$1.44\times 10^{-57}$	0.179	$2.76\times 10^{-3}$	+5.13% ($p=9.70\times 10^{-4}$)	$1.39\times 10^{-3}$
8	−0.579	$2.59\times 10^{-26}$	0.304	$2.44\times 10^{-7}$	+14.45% ($p=4.88\times 10^{-11}$)	$1.63\times 10^{-10}$
9	−0.369	$2.06\times 10^{-10}$	0.388	$1.97\times 10^{-11}$	+20.07% ($p=8.15\times 10^{-20}$)	$8.15\times 10^{-19}$
10	−0.630	$4.28\times 10^{-32}$	0.218	$2.56\times 10^{-4}$	+8.66% ($p=1.43\times 10^{-5}$)	$2.38\times 10^{-5}$

(2.4) $$\log(\lambda_{ik,\mathrm{raw}})=a_{k}+b_{k}\log(S_{i})+c_{k}\;{\mathrm{sex}}_{i}+\varepsilon_{i},$$

and the descriptive size-normalized value was calculated as

(2.5) $$\lambda_{ik,\mathrm{scaled}}=\lambda_{ik,\mathrm{raw}}{\left(\frac{S_{i}}{S_{0}}\right)}^{\gamma_{k}},\;\gamma_{k}=-b_{k}.$$

For modes 1–10, $\gamma_{k}$ was 1.382, 1.834, 1.676, 2.393, 2.068, 2.139, 2.346, 2.326, 1.887 and 2.136, respectively.

Adjusted sex effects were estimated from

(2.6) $$\log(\lambda_{\mathrm{raw}})∼\log(\mathrm{scale})+\mathrm{sex}+\mathrm{age}.$$

The sex coefficient was expressed as the percentage difference in eigenvalue for male relative to female pelves. Benjamini–Hochberg false-discovery-rate adjusted q-values are reported across the 10 mode-wise tests.

To assess whether individual conventional pelvimetric dimensions explained modal stiffness beyond pelvic scale and age, we fitted female-only models for modes 9 and 2:

(2.7) $$\log(\lambda_{\mathrm{raw}})∼\log(\mathrm{scale})+\mathrm{age}+\mathrm{dimension}.$$

The restriction to females reflects the obstetric context in which the classical pelvic types are conventionally applied. The incremental contribution of each dimension was reported as partial $R^{2}$.

Modal participation fractions were summarized as cohort means because they are compositional quantities. Response concentration was also described by the effective number of participating modes,

(2.8) $$N_{\mathrm{eff}}=\exp\left(-\sum_{i}p_{i}\log p_{i}\right),$$

where $p_{i}$ is the energy fraction of mode i after normalization over modes 1–10. Larger values indicate a more distributed modal response.

## 3. Results

### 3.1. Cohort differences in scale and pelvic dimensions

The cohort comprised 278 individuals, including 150 female and 128 male individuals. Age did not differ significantly between the sexes (table 2). Female pelves had smaller overall scale than male pelves, but larger AP and PIT dimensions, a wider subpubic angle, and greater biischiadic and bituberous widths. Sacral width did not differ significantly between the sexes.

**Table 2.** Cohort summary and static pelvic measures. Values are mean ± s.d.; p-values are Welch two-sample t-tests (female versus male). AP, anteroposterior inlet diameter; PIT, pelvic inlet transverse diameter.

measure	female (n=150)	male (n=128)	p
age (years)	53.4±19.7	54.5±18.9	0.617
scale	0.925±0.045	0.998±0.045	$6.51\times 10^{-32}$
effective thickness (mm)	4.96±0.36	5.51±0.36	$4.44\times 10^{-29}$
effective density (kg m${}^{-3}$)	1341±79	1321±74	0.028
AP (mm)	121.0±10.0	112.3±9.1	$4.63\times 10^{-13}$
PIT (mm)	134.9±8.6	127.7±7.6	$2.88\times 10^{-12}$
subpubic angle (deg)	85.8±6.1	69.1±5.3	$7.30\times 10^{-71}$
biacetabular width (mm)	129.1±8.2	122.0±7.2	$2.94\times 10^{-13}$
biischiadic width (mm)	109.9±7.1	97.7±7.1	$9.40\times 10^{-35}$
bituberous width (mm)	136.5±8.3	127.3±7.5	$3.03\times 10^{-19}$
sacral width (mm)	116.7±5.7	117.5±6.5	0.327
iliopectineal eminence width (mm)	106.2±7.0	97.9±6.0	$1.90\times 10^{-22}$

Because scale was defined from total pelvic volume, the volume-scale relation is definitional and is not reported as an empirical allometry. Among the remaining quantities, total surface area scaled sub-isometrically with pelvic scale (slope =1.684, $R^{2}=0.894$), total mass increased with scale (slope =2.679, $R^{2}=0.902$), effective thickness increased with scale (slope =1.316, $R^{2}=0.837$) and effective density declined modestly with scale (slope =−0.321, $R^{2}=0.117$; table 3).

**Table 3.** Empirical allometric scaling of geometric and material-derived quantities. Slopes are from log–log regressions against pelvic scale. Total volume is omitted because pelvic scale was defined from volume.

outcome versus scale	slope	p	$r^{2}$
total surface area	1.684	$2.53\times 10^{-136}$	0.894
total mass	2.679	$2.58\times 10^{-141}$	0.902
effective thickness	1.316	$1.25\times 10^{-110}$	0.837
effective density	−0.321	$5.07\times 10^{-9}$	0.117

### 3.2. Scale dependence, adjusted sex effects and pelvimetric associations

Before allometric size normalization, log pelvic scale was negatively correlated with log eigenvalue for all 10 modes (table 1 and figure 2). After normalization to the cohort geometric-mean scale, the correlation was near zero for modes 1 and 5 and positive for several other modes. The strongest positive scaled correlations were observed for modes 2 and 9.

In models using raw eigenvalues with scale and age as covariates, the largest adjusted sex effects occurred in modes 2 and 9. Male pelves had eigenvalues 20.72% higher than female pelves in mode 2 and 20.07% higher in mode 9. Positive adjusted male effects were also present in modes 3, 4, 7, 8 and 10. Mode 1 showed no detectable sex effect, and modes 5 and 6 did not survive the same threshold of evidence (table 1).

In female-only pelvimetry models, AP was the strongest single predictor of mode 9 stiffness after adjustment for scale and age (partial $R^{2}=0.239$, $p=3.04\times 10^{-10}$), followed by PIT (partial $R^{2}=0.127$, $p=8.82\times 10^{-6}$). Iliopectineal eminence width and sacral width showed similar but smaller contributions (table 4 and figure 3). These significant inlet-related associations were negative, indicating lower mode 9 eigenvalues with larger AP, PIT, iliopectineal eminence width or sacral width; subpubic angle showed a smaller positive association. For mode 2, the largest partial $R^{2}$ was observed for bituberous width (partial $R^{2}=0.102$, $p=7.80\times 10^{-5}$), followed by PIT, biischiadic width and biacetabular width, all with negative coefficients. No pelvimetric variable had a partial $R^{2}$ above 0.239 for either mode.

**Figure 3.**
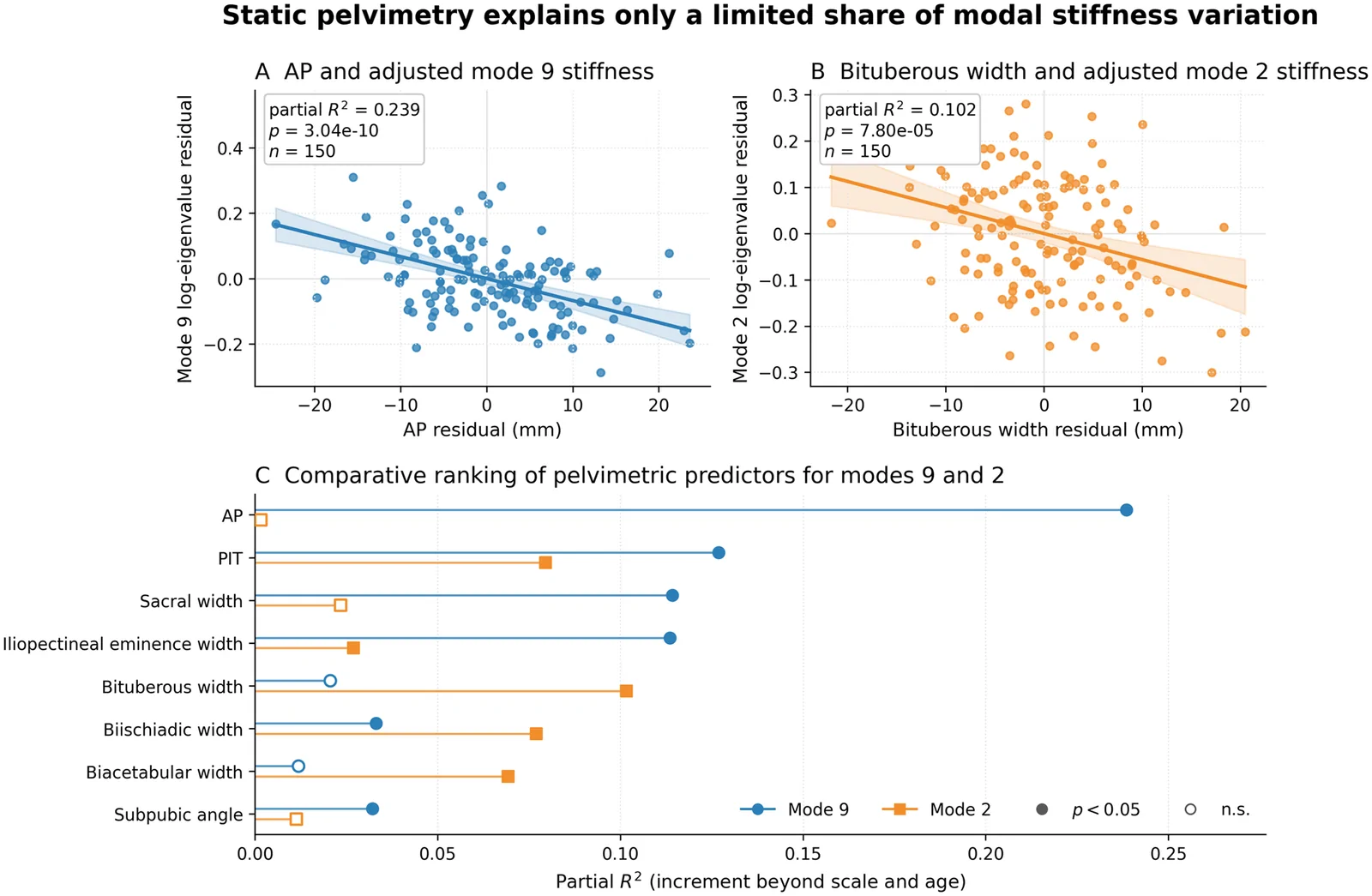
(A) Added-variable plot showing the adjusted association between the anteroposterior inlet diameter (AP) and mode 9 stiffness, expressed as the log-transformed eigenvalue. (B) Added-variable plot showing the adjusted association between bituberous width and mode 2 stiffness. (C) Comparative ranking of pelvimetric predictors for modes 9 and 2, expressed as partial $R^{2}$ values, i.e. the incremental proportion of variance explained by each pelvimetric variable beyond scale and age.

**Table 4.** Female-only associations between static pelvic dimensions and modal stiffness for the two most sex-biased modes. Values are partial $R^{2}$ and p for models of the form $\log(\lambda_{\mathrm{raw}})∼\log(\mathrm{scale})+\mathrm{age}+\mathrm{dimension}$.

	mode 9		mode 2	
dimension	partial $r^{2}$	p	partial $r^{2}$	p
AP	0.239	$3.04\times 10^{-10}$	0.002	0.628
PIT	0.127	$8.82\times 10^{-6}$	0.079	$5.18\times 10^{-4}$
iliopectineal eminence width	0.114	$2.83\times 10^{-5}$	0.027	0.046
sacral width	0.114	$2.70\times 10^{-5}$	0.023	0.063
biacetabular width	0.012	0.188	0.069	$1.23\times 10^{-3}$
biischiadic width	0.033	0.027	0.077	$6.43\times 10^{-4}$
bituberous width	0.021	0.082	0.102	$7.80\times 10^{-5}$
subpubic angle	0.032	0.029	0.011	0.197

### 3.3. Modal participation under support and opening probes

Under bilateral stance, mode 2 accounted for most of the response in both sexes (84.6% in female pelves and 84.5% in male pelves; table 5). Under unilateral stance, modes 1–3 together accounted for 92.7% of the response in female pelves and 91.7% in male pelves. Sex differences in support-loading participation fractions were small in comparison with the dominant mode contributions. The corresponding modal decompositions across all loading probes are shown in figure 4.

**Figure 4.**
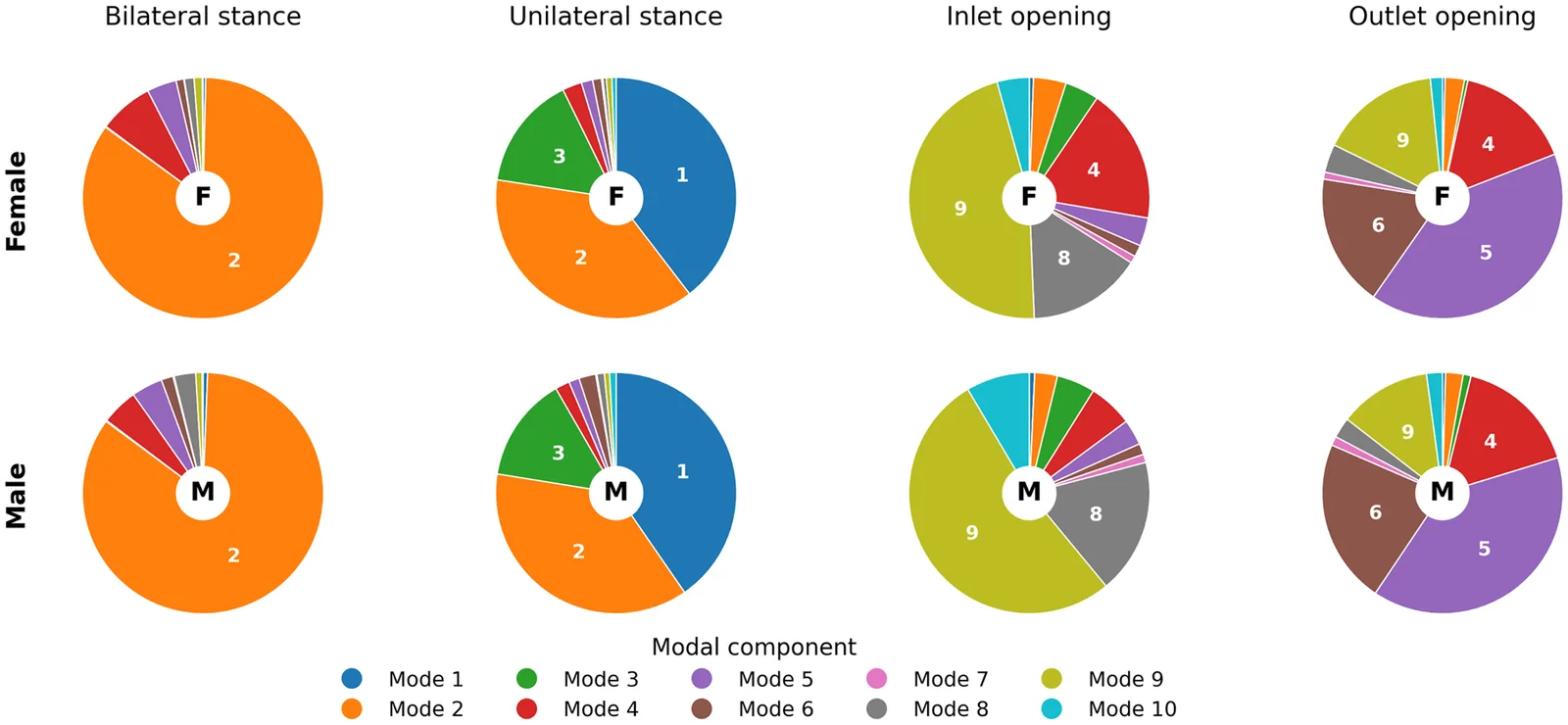
Fractional modal decomposition across standardized subject-specific loading probes. Each small pie chart shows the cohort mean contribution of modes 1–10 to the modal response. Segment colours denote individual modal components (modes 1–10), and labels within selected sectors indicate dominant modes.

**Table 5.** Mean modal participation fractions (%) for standardized subject-specific loading probes after normalization over modes 1–10. Values are descriptive cohort means for compositional modal fractions. The first 10 modes retained >99% of total response energy in the support scenarios and 91–99% in the inlet and outlet probes.

scenario	sex	M1	M2	M3	M4	M5	M6	M7	M8	M9	M10
bilateral stance	female	0.4	84.6	0.1	7.3	4.0	1.0	0.1	1.3	1.1	0.1
bilateral stance	male	0.6	84.5	0.1	4.9	4.2	1.6	0.2	2.9	0.8	0.1
unilateral stance	female	39.6	37.8	15.3	2.6	1.5	1.2	0.2	0.5	0.7	0.6
unilateral stance	male	40.4	37.2	14.1	1.9	1.4	2.3	0.2	1.0	0.7	0.9
inlet opening	female	0.6	4.4	4.5	18.2	3.8	1.6	1.0	15.3	46.4	4.3
inlet opening	male	0.7	3.1	5.1	6.0	3.4	1.5	1.1	18.1	52.5	8.5
outlet opening	female	0.4	2.6	0.4	15.7	40.7	17.8	0.9	3.8	16.1	1.6
outlet opening	male	0.4	2.4	1.1	16.4	39.2	22.1	1.2	2.8	12.4	2.1

Under inlet opening, mode 9 had the largest response fraction in 265 of 278 subjects. The mean mode 9 fraction was 46.4% in female pelves and 52.5% in male pelves. Female pelves had greater mean participation of mode 4 than male pelves (18.2% versus 6.0%), whereas mode 8 contributed substantially in both sexes (15.3% and 18.1%). The effective number of participating modes was higher in female than male pelves for inlet opening ($N_{\mathrm{eff}}=4.53\pm 0.71$ versus 4.04±0.76; Welch $p=8.05\times 10^{-8}$).

Under outlet opening, mode 5 had the largest mean fraction in both sexes (40.7% in female pelves and 39.2% in male pelves). Female pelves had greater concurrent participation of mode 9 (16.1% versus 12.4%), whereas male pelves had greater participation of mode 6 (22.1% versus 17.8%). Representative load applications and modal deformation patterns are shown in figure 5.

**Figure 5.**
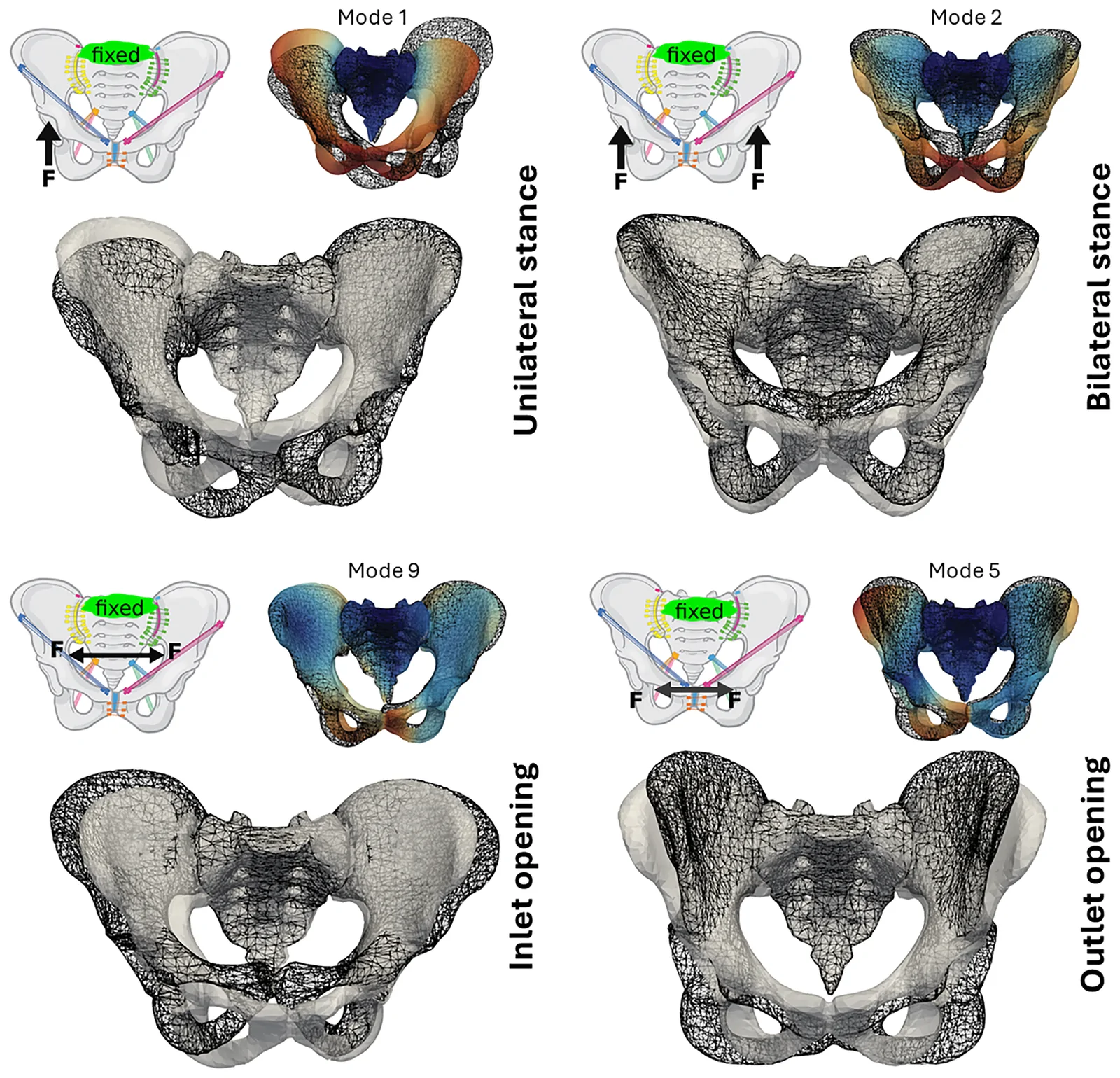
Representative modal deformation patterns associated with the four standardized loading probes. In each schematic inset, black arrows labelled F indicate the directions of the applied total-force vectors, the green region marks the restrained superior S1 surface, and coloured lines identify the represented ligament groups. The adjacent coloured pelvis shows the single eigenmode with the largest mean participation under that probe; colours encode relative nodal displacement magnitude within the mode. Single modes can include shear-dominated deformation and may therefore display an asymmetric displacement-magnitude pattern despite symmetric loading. The complete probe response is reconstructed from the weighted combination of all participating modes; the wireframe overlays illustrate the dominant deformation pathway.

### 3.4. Soft-tissue sensitivity profiles across modes

Soft-tissue sensitivity profiles were compared for modes 2, 3, 4, 8 and 9, with left and right structures averaged for bilateral tissues (table 6). Ligament and solid-region results are reported as separate within-class rankings because the two sensitivity measures have different units and perturbation definitions.

**Table 6.** Soft-tissue sensitivity profiles for selected modes. Values are cohort means from template-defined local perturbation operators, with bilateral structures averaged. Percentages are within-class contributions for each mode, calculated separately for solid-region and ligament sensitivities because these measures have different definitions.

structure	mode 2	mode 3	mode 4	mode 8	mode 9
*solid-region sensitivity*					
SIJ cartilage	$2.10\times 10^{-4}$ (95.7%)	$2.69\times 10^{-4}$ (40.3%)	$6.08\times 10^{-4}$ (67.6%)	$2.01\times 10^{-3}$ (93.5%)	$3.00\times 10^{-3}$ (59.2%)
pubic symphysis	$9.45\times 10^{-6}$ (4.3%)	$3.99\times 10^{-4}$ (59.7%)	$2.92\times 10^{-4}$ (32.4%)	$1.39\times 10^{-4}$ (6.5%)	$2.07\times 10^{-3}$ (40.8%)
*ligament sensitivity*					
symphyseal ligament group	$2.12\times 10^{-9}$ (5.7%)	$2.96\times 10^{-8}$ (30.4%)	$7.56\times 10^{-8}$ (38.3%)	$7.86\times 10^{-8}$ (15.8%)	$1.09\times 10^{-6}$ (59.3%)
anterior SIJ ligaments	$2.59\times 10^{-8}$ (69.2%)	$4.68\times 10^{-8}$ (48.0%)	$8.38\times 10^{-8}$ (42.4%)	$2.87\times 10^{-7}$ (57.7%)	$4.64\times 10^{-7}$ (25.3%)
interosseous SIJ ligaments	$4.68\times 10^{-11}$ (0.1%)	$3.75\times 10^{-10}$ (0.4%)	$1.20\times 10^{-8}$ (6.1%)	$2.20\times 10^{-8}$ (4.4%)	$2.40\times 10^{-7}$ (13.1%)
posterior SIJ ligaments	$2.87\times 10^{-9}$ (7.7%)	$7.06\times 10^{-9}$ (7.2%)	$2.05\times 10^{-8}$ (10.4%)	$3.76\times 10^{-8}$ (7.6%)	$1.27\times 10^{-8}$ (0.7%)
sacrospinous ligaments	$5.85\times 10^{-9}$ (15.6%)	$1.23\times 10^{-8}$ (12.6%)	$4.76\times 10^{-9}$ (2.4%)	$1.75\times 10^{-8}$ (3.5%)	$6.57\times 10^{-9}$ (0.4%)
sacrotuberous ligaments	$6.49\times 10^{-10}$ (1.7%)	$1.27\times 10^{-9}$ (1.3%)	$8.90\times 10^{-10}$ (0.5%)	$5.48\times 10^{-8}$ (11.0%)	$2.37\times 10^{-8}$ (1.3%)

Among the analysed modes, total solid-region sensitivity was highest in mode 9, followed by mode 8. In mode 9, the SIJ cartilage accounted for 59.2% of solid-region sensitivity and the pubic symphysis for 40.8%. Mode 8 solid-region sensitivity was concentrated mainly at the SIJ cartilage (93.5%).

Total ligament sensitivity was also highest in mode 9. Within the mode 9 ligament profile, the largest contribution came from the symphyseal ligament group (59.3%), followed by the anterior SIJ ligaments (25.3%) and interosseous SIJ ligaments (13.1%). Mode 8 had a lower total ligament sensitivity and was dominated by anterior SIJ ligaments (57.7%). Modes 2–4 had lower total ligament sensitivities, with contributions mainly from the anterior SIJ and symphyseal ligament groups.

## 4. Discussion

### 4.1. A selective mechanical phenotype of pelvic dimorphism

Read together, the results shift pelvic dimorphism from a purely geometric description to a mechanical one. The cohort showed the expected sex differences in inlet dimensions, subpubic angle and outlet-related breadths, but those differences were not a simple uniform rescaling of the pelvis. The modal analysis adds the missing mechanical layer: scale explained much of the background spectrum, yet selected deformation pathways remained sexually structured after scale and age were accounted for.

This selectivity is central. If pelvic dimorphism were only an effect of size, the adjusted modal spectrum should have been largely neutral. Instead, modes 2 and 9 remained the clearest sex-biased pathways, with additional effects in several neighbouring modes. The result is therefore not that the whole female pelvis is globally softer, nor that the whole male pelvis is globally stiffer. Rather, sex differences are concentrated in particular deformation families within a shared pelvic-ring architecture.

### 4.2. Static diameters are predictors, not the mechanical phenotype itself

The pelvimetry results show why geometry remains important but incomplete. AP and PIT were related to mode 9, and bituberous width was related to mode 2, so conventional metric dimensions clearly carry mechanical information. At the same time, no single dimension captured most of the modal variance. A diameter describes bony space along one line; an eigenmode reflects how the full ring deforms under the combined influence of shape, bone distribution, cartilage regions and ligamentous constraints.

This distinction is especially important for obstetric interpretation. Static pelvimetry can describe the canal, but it cannot by itself describe how the pelvic ring accesses deformation pathways when loaded. The present modal approach therefore does not replace pelvimetry. It explains why similar dimensions need not imply identical compliance, and why mechanically relevant compliance may be concentrated in specific deformation pathways rather than expressed as global pelvic softening.

### 4.3. Shared support, selective opening and anterior constraint

The load projections clarify how these pathways are used under standardized probe fields. Bilateral and unilateral stance recruited a common low-order support pattern in both sexes, whereas inlet opening exposed a different organization of response energy. Mode 9 was the largest inlet-opening component in almost all subjects, but male pelves concentrated more of the response into this one mode. Female pelves showed a higher effective number of participating modes, indicating that the inlet-opening response was spread across a broader set of coordinated pathways.

This is a more precise interpretation than saying that female pelves are simply more compliant. The difference lies in the organization of compliance: support mechanics remain broadly shared, while opening probes redistribute the response in a sex-specific way. Outlet opening supports a less evident, but similar view because mode 5 dominated in both sexes, yet female pelves retained greater concurrent mode 9 participation. Inlet and outlet mechanics therefore appear to be overlapping expressions of one constrained ring rather than independent modules.

The sensitivity results provide the anatomical link. Mode 9 combined the highest solid-region sensitivity with a ligament profile dominated by the symphyseal ligament group, anterior SIJ ligaments and interosseous SIJ ligaments. This pattern is consistent with clinical and anatomical evidence that pregnancy- and childbirth-related mobility is concentrated around the pubic symphysis and anteriorly located sacroiliac constraints [18–21], while posterior ligaments such as the sacrospinous and sacrotuberous ligaments contribute importantly to posterior pelvic stability [22,23]. The present results extend that interpretation by suggesting that soft-tissue influence on pelvic mechanics may be mode-selective rather than global.

### 4.4. Clinical relevance: pathway-selective laxity as a testable mechanism

Clinically, these findings provide a mechanistic vocabulary for conditions often discussed under the broad term pelvic laxity. Pregnancy-related pelvic girdle pain, symphyseal separation and SIJ-related dysfunction can involve different anatomical regions and different loading tasks [18,24–26]. In the present models, the same soft-tissue constraints did not contribute uniformly to all modes. Anterior SIJ and symphyseal constraints had disproportionate influence on the inlet-opening pathway, while support loading remained organized around a shared low-order backbone.

This suggests a clinically testable hypothesis: changes in ligamentous or cartilaginous properties during pregnancy and parturition may selectively alter access to particular deformation pathways rather than simply increasing global pelvic mobility. A small change in symphyseal or anterior SIJ constraint could increase access to an opening pathway without equivalently compromising stance mechanics. Future work can test this pathway-level interpretation by combining pregnancy status, pain phenotype, dynamic imaging, fetal presentation and delivery outcome with subject-specific mechanical models.

### 4.5. Scope of the obstetric interpretation and limitations

The claim of the present study is focused: both sexes share a support-oriented low-order repertoire, but differ in the accessibility and recruitment of selected deformation pathways, especially the inlet-opening pathway represented by mode 9. In this framework, the relevant phenotype is not pelvic size, canal shape or laxity alone, but the organization of mode-specific compliance within a constrained ring.

Several limitations define the scope of this interpretation. The cohort represents a Central European clinical CT sample and was not linked to obstetric outcomes. The finite-element formulation captures the small-deformation elastic regime, uses phantomless CT-derived material descriptors and omits subject-specific nonlinear tissue behaviour. The mechanical probes used standardized template-derived anatomical definitions for supports and loading directions, which improves comparability but does not capture all individual variation in physiological loading. They therefore characterize comparative pathway recruitment rather than time-dependent gait mechanics. Dynamic gait modelling is feasible in principle, but applying it consistently across 278 subject-specific pelves would require a standardized kinematic and loading workflow and likely substantial model simplification. Nonlinear, tension-only or volumetric ligament formulations and hyperelastic, viscoelastic or contact-based representations of the cartilage and pubic symphysis could alter the tangent stiffness, local load transfer, absolute eigenvalues and potentially the spacing or ordering of nearby modes. Conversely, the local sensitivity analysis used template-defined perturbation operators and should be interpreted as a ranking of constraint influence rather than as a full subject-specific perturbation study.

## 5. Conclusion

In this population-scale, correspondence-preserving analysis of 278 human pelves, allometry explained much of the baseline accessibility of deformation pathways but did not exhaust pelvic mechanics. After adjustment for scale and age, strong sex differences remained in selected modes, especially modes 2 and 9. Habitual support scenarios relied on a shared low-order modal backbone in both sexes, whereas inlet- and outlet-opening probes revealed sex-specific reorganization of compliance. The principal inlet-opening pathway was most sensitive to the pubic symphysis, anterior SIJ ligaments and sacroiliac regions.

The key novelty is therefore not the rediscovery of pelvic dimorphism, but the demonstration that pelvic function is organized through mode-specific compliance that cannot be reduced to size or to any single obstetric diameter. Within the limits of the present elastic modelling framework, female pelves in this cohort are best interpreted as pathway-selective compliant rings: structures that preserve ordinary support while maintaining selectively accessible, anteriorly constrained pathways relevant to childbirth mechanics and pelvic-ring clinical disorders.

## Data Availability

The source BoneDat morphology dataset is available through the published BoneDat data descriptor and associated repository. Processed derived datasets supporting the present analyses, together with analysis scripts, simulation code, model-parameter files, figure-generation scripts and a data dictionary, are deposited in Figshare [27]. The shared files do not include the original clinical CT scans.

## Declaration of AI use

During manuscript preparation, AI-assisted tools were used for language editing, structural checks and preparation of submission-readiness recommendations. All scientific content, analyses, interpretations and final text were reviewed and approved by the authors, who take full responsibility for the manuscript. The AI-assisted tools were used under a business licence with restricted data-use settings, including restrictions on the use of submitted content for model training.
